# Implementing an Enhanced Recovery after Surgery (ERAS) Protocol Reduces Intra-Operative Opiate Use in Outpatient Breast Surgery

**DOI:** 10.1093/asjof/ojaf018.006

**Published:** 2025-05-13

**Authors:** Lauren Catterall, Danielle Olla, Ashley-Ann A Walker, Molly Smith, Kristin Delfino, Sharon Kim, Alaine Nixon, Nicole Sommer

**Affiliations:** SIU School of Medicine, Springfield, IL; Moffitt Cancer Center, Tampa, FL; SIU School of Medicine, Springfield, IL; SIU School of Medicine, Springfield, IL; SIU School of Medicine, Springfield, IL; SIU School of Medicine, Springfield, IL; SIU School of Medicine, Springfield, IL; SIU School of Medicine, Springfield, IL

## Abstract

**Goals/Purpose:**

The enhanced recovery after surgery (ERAS) protocol has been demonstrated to reduce post-operative opioid prescriptions following breast reconstruction procedures. There is emerging evidence that ERAS protocols are efficacious for outpatient breast surgery, diminishing PACU opiate use, decreasing post operative nausea and vomiting, and decreasing outpatient opiate prescriptions. However, whether ERAS protocols impact intra-operative opiate use for outpatient breast surgeries is unknown. The purpose of this investigation was to determine the impact of ERAS protocols on intra-operative opiate use for outpatient breast surgery procedures and validate its utility in post-operative pain management for outpatient breast surgery.

**Methods/Technique:**

A retrospective chart review was conducted of patients receiving outpatient breast surgery by a single surgeon at our institution four years prior to instituting the ERAS protocol in May of 2015 (pre-ERAS) and four years after instituting the ERAS protocol in May of 2019 (post-ERAS). The protocol included preoperative hydration, pectoral nerve blocks, peri-operative Gabapentin 200-300mg TID, Acetaminophen 1000 QID, Celecoxib 200-400mg BID or Ibuprofen 800mg TID and optional Orphenadrine 100mg BID for up to 7 days. Patients also received an educational component involving a supplemental brochure and discussions during their clinic visits.

The intra-operative and PACU opiate use was obtained from anesthesia and hospital records. The type, route, and amount of opiate was documented and converted to oral morphine milligram equivalents (MME). The intra-operative and PACU opiate use and MMEs were compared between the two groups.

**Results/Complications:**

A total of 316 patients were included in the pre-ERAS cohort and 257 patients were included in the post-ERAS cohort. Outpatient surgeries included breast reductions, secondary revision procedures for breast reconstruction including symmetry procedures, breast augmentation, primary breast reconstruction, and implant exchange. Post-ERAS patients received 30% fewer MMEs intraoperatively (50.9 MMEs vs 70.8 MMEs, p<0.0001). The post-ERAS cohort also received significantly fewer opiates in PACU (19.1 MMEs vs 30.3 MMEs, p<0.0001) and 30% of patients did not received any opiates in PACU. Although there were no significant differences between the two groups in the number of patients receiving IV medications such as fentanyl, hydromorphone, or morphine, fewer post-ERAS patients received oral opiates (p<0.0001).

**Conclusion:**

This study validates prior literature demonstrating patients receiving ERAS protocols for outpatient breast surgery receive fewer opiates in PACU. This difference is attributable to a decrease in long-acting oral opiate prescriptions rather than acute onset intravenous (IV) medications frequently used in phase I of anesthesia. ERAS also significantly decreases intra-operative opiate administration which can be useful in diminishing post-operative nausea and vomiting

